# Trimethoprim-sulfamethoxazole versus trimethoprim-sulfamethoxazole plus doxycycline as oral eradicative treatment for melioidosis (MERTH): a multicentre, double-blind, non-inferiority, randomised controlled trial

**DOI:** 10.1016/S0140-6736(13)61951-0

**Published:** 2014-03-01

**Authors:** Ploenchan Chetchotisakd, Wirongrong Chierakul, Wipada Chaowagul, Siriluck Anunnatsiri, Kriangsak Phimda, Piroon Mootsikapun, Seksan Chaisuksant, Jiraporn Pilaikul, Bandit Thinkhamrop, Sunchai Phiphitaporn, Wattanachai Susaengrat, Chalongchai Toondee, Surasakdi Wongrattanacheewin, Vanaporn Wuthiekanun, Narisara Chantratita, Janjira Thaipadungpanit, Nicholas P Day, Direk Limmathurotsakul, Sharon J Peacock

**Affiliations:** a Faculty of Medicine, Khon Kaen University, Thailand; b Melioidosis Research Centre, Khon Kaen University, Thailand; c Faculty of Tropical Medicine, Mahidol University, Thailand; d Sappasithiprasong Hospital, Ubon Ratchathani, Thailand; e Udon Thani Hospital, Udon Thani, Thailand; f Khon Kaen Hospital, Khon Kaen, Thailand; g Mahasarakam Hospital, Mahasarakam, Thailand; h Faculty of Public Health, Khon Kaen University, Thailand; i University of Oxford, Churchill Hospital, Oxford, UK; j University of Cambridge, Addenbrooke's Hospital, Cambridge, United Kingdom

## Abstract

**Background:**

Melioidosis, an infectious disease caused by the Gram-negative bacillus *Burkholderia pseudomallei*, is difficult to cure. Antimicrobial treatment comprises intravenous drugs for at least 10 days, followed by oral drugs for at least 12 weeks. The standard oral regimen based on trial evidence is trimethoprim-sulfamethoxaxole (TMP-SMX) plus doxycycline. This regimen is used in Thailand but is associated with side-effects and poor adherence by patients, and TMP-SMX alone is recommended in Australia. We compared the efficacy and side-effects of TMP-SMX with TMP-SMX plus doxycycline for the oral phase of melioidosis treatment.

**Methods:**

For this multi-centre, double-blind, non-inferiority, randomised placebo-controlled trial, we enrolled patients (aged ≥15 years) from five centres in northeast Thailand with culture-confirmed melioidosis who had received a course of parenteral antimicrobial drugs. Using a computer-generated sequence, we randomly assigned patients to receive TMP-SMX plus placebo or TMP-SMX plus doxycycline for 20 weeks (1:1; block size of ten, stratified by study site). We followed patients up every 4 months for 1 year and annually thereafter to the end of the study. The primary endpoint was culture-confirmed recurrent melioidosis, and the non-inferiority margin was a hazard ratio (HR) of 1·7. This study is registered with www.controlled-trials.com, number ISRCTN86140460.

**Findings:**

We enrolled and randomly assigned 626 patients: 311 to TMP-SMX plus placebo and 315 to TMP-SMX plus doxycycline. 16 patients (5%) in the TMP-SMX plus placebo group and 21 patients (7%) in the TMP-SMX plus doxycycline group developed culture-confirmed recurrent melioidosis (HR 0·81; 95% CI 0·42–1·55). The criterion for non-inferiority was met (p=0.01). Adverse drug reactions were less common in the TMP-SMX plus placebo group than in the TMP-SMX plus doxycycline group (122 [39%] *vs* 167 [53%]).

**Interpretation:**

Our findings suggest that TMP-SMX is not inferior to TMP-SMX plus doxycycline for the oral phase of melioidosis treatment, and is preferable on the basis of safety and tolerance by patients.

**Funding:**

Thailand Research Fund, the Melioidosis Research Center, the Center of Excellence in Specific Health Problems in Greater Mekong Sub-region cluster, and the Wellcome Trust.

## Introduction

Melioidosis is a serious infection caused by the Gram-negative bacillus *Burkholderia pseudomallei*, found in soil and water.^1^ The reported incidence of human melioidosis is highest in northeast Thailand (crude case fatality rate 43%) and northern Australia (14%).^2^, ^3^ Melioidosis also affects travellers to melioidosis-endemic regions of the world,^4^ which includes much of Asia, regions of South America, various Pacific and Indian Ocean islands, and some countries in Africa including Nigeria, Gambia, Kenya, and Uganda.^1^ First-line initial antimicrobial treatment is parenteral ceftazidime or a carbapenem drug (either meropenem or imipenem) for at least 10 days.^5^ Patients are then switched to oral antimicrobials for at least 12 weeks. This extended period of treatment compared with most other bacterial infections is needed to achieve cure and prevent recurrent infection,^5^ which has been reported to occur in 16% of cases within 10 years of the primary infection and has a case fatality rate of 24% in Thailand.^6^

The recommended oral antimicrobial regimen for melioidosis in Thailand is trimethoprim-sulfamethoxazole (TMP-SMX) plus doxycycline. This recommendation is based on findings that this regimen is as effective as, and better tolerated than, the previously recommended regimen of TMP-SMX plus doxycycline and chloramphenicol.^7^ However, a quarter of patients with melioidosis given TMP-SMX plus doxycycline develop an adverse drug reaction.^7^ Such adverse reactions often results in a switch to second-line treatment (usually amoxicillin-clavulanic acid), which is strongly associated with an increased risk of relapse.^6^ Findings from a descriptive 10 year cohort study done in Australia reported recurrent infection in less than 2% of patients (one of 60 patients) who had oral treatment with TMP-SMX alone;^8^ TMP-SMX has since become the standard regimen in Australia^3^ and is occasionally used in Thailand.^9^ We proposed that TMP-SMX alone was an adequate treatment for melioidosis, and did a clinical trial to compare the efficacy and safety of TMP-SMX versus TMP-SMX plus doxycycline for the oral treatment phase of melioidosis.

## Methods

### Study design and participants

We did this multicentre, double-blind, non-inferiority, randomised placebo-controlled trial in five hospitals in northeast Thailand: Sappasithiprasong Hospital (Ubon Ratchathani), Srinagarind Hospital (Khon Kaen), Udon Thani Hospital (Udon Thani), Mahasarakam Hospital (Mahasarakam), and Khon Kaen Hospital (Khon Kaen). We enrolled adult patients (aged ≥15 years) with culture-confirmed melioidosis who had been satisfactorily treated with parenteral antimicrobials, or who had mild localised disease that was not considered to need intravenous antimicrobial treatment by the attending physicians. We defined satisfactory clinical improvement from parenteral treatment as cessation of fever for at least 48 h and the ability to take oral drugs. We excluded patients if they were infected by *B pseudomallei* that was resistant to TMP-SMX or doxycycline, if their melioidosis infection was recurrent (defined as having a previous episode of culture-confirmed melioidosis within the past 2 years), or if they had a contraindication to either TMP-SMX or doxycycline (pregnancy, lactation, glomerular filtration rate <15 mL/min estimated by the Cockcroft-Gault equation, aspartate aminotransferase more than five times the upper limit of normal, alanine aminotransferase more than five times the upper limit of normal, known glucose-6-phosphase dehydrogenase deficiency, or history of hypersensitivity to TMP-SMX, doxycycline, or both). Resistance to doxycycline was determined by disc diffusion as an inhibition zone diameter ≤12 mm, which was modified from the Clinical and Laboratory Standards Institute (CLSI) breakpoint recommended for Enterobacteriaceae*.*^10^, ^11^ Resistance to TMP-SMX was determined by Etest (AB Biodisk, Solna, Sweden) as a minimum inhibitory concentration of 4/76 mg/L or higher, which was modified from the CLSI breakpoint for *B pseudomallei* determined by broth dilution method.^11^, ^12^

The trial was done in accordance with the principles of good clinical practice, and the ethical principles in the Declaration of Helsinki. The study protocol was approved by the local ethical committees and the institutional review boards of all participating hospitals. The study was reviewed by an independent data safety and monitoring board. All patients gave signed or fingerprinted informed consent before randomisation. This trial is registered with www.controlled-trials.com, number IRSCTN86140460.

### Randomisation and masking

We randomly allocated patients in a 1:1 ratio to receive TMP-SMX with either placebo doxycycline (hereafter referred to as placebo) or doxycycline, which were identical in appearance. Randomisation and masking was done at the coordinating centre at the Mahidol-Oxford Tropical Medicine Research Unit (Bangkok, Thailand). The allocation sequence was computer generated with a block size of ten and was stratified by study site. To achieve treatment concealment, TMP-SMX and either placebo or doxycycline were dispensed into sequential, identical, tamper-proof bottles for each participant for 20 weeks. The study drug bottles were labelled with sequential code numbers and distributed to the study sites. TMP-SMX, placebo, and doxycycline were manufactured and provided by the Siam Pharmaceutical Company (Bangkok, Thailand). All patients and study investigators were unaware of the drug allocation throughout the study. The randomisation codes remained sealed until after data collection, data cleaning, and completion of a masked analysis.

### Procedures

All patients received TMP-SMX plus placebo or TMP-SMX plus doxycycline for a minimum of 20 weeks. TMP-SMX (80 mg TMP and 400 mg SMX) tablets were prescribed using a weight-based dosage, as follows: bodyweight less than 40 kg or estimated glomerular filtration rate 15–29 mL/min, 160 mg TMP and 800 mg SMX twice daily; bodyweight of 40 kg to 60 kg, 240 mg TMP and 1200 mg SMX twice daily; and bodyweight greater than 60 kg, 320 mg TMP and 1600 mg SMX twice daily.^13^ Doxycycline or placebo was prescribed as a 100 mg tablet to be taken twice daily. Patients were advised to repeat the dose if vomiting occurred within 30 min of their taking the tablet. The minimum duration of 20 weeks was based on a combination of current practice and available evidence. The recommended duration for oral antimicrobials is 12–20 weeks in Thailand,^6^ and 3–6 months in Australia,^14^ a discrepancy that shows the uncertainty about the optimum duration. Findings from a retrospective study in Thailand showed that treatment for longer than 12 weeks was associated with lower risk of relapse.^6^ We therefore chose to use 20 weeks as a minimum duration rather than 12 weeks or an empirically chosen point between the two.

After enrolment, we followed patients up at weeks 4, 12, and 20 of oral treatment, every 4 months for 1 year after completion of treatment, and annually thereafter to the end of the study. Patients who did not attend scheduled appointments were contacted by telephone. The trial was designed to stop 1 year after the last participant was enrolled. At each clinical visit, we undertook a clinical examination and laboratory analyses, including complete blood count, blood sugar, blood urea nitrogen, creatinine, electrolyte, and liver function tests. Chest radiography and abdominal ultrasonography were done at enrolment, and repeated at weeks 12 and 20 if an abnormality was detected on the first test. We asked patients to bring the study drug bottles to follow-up visits, at which drug compliance was checked by pill counts. Treatment with the randomised drugs was extended beyond 20 weeks if clinically indicated because of evidence of residual infection, as decided by the treating physician. Concealed study drug bottles labelled with unique spare sequential code numbers were separately prepared for patients who needed treatment for more than 20 weeks. As needed, the site investigator contacted the coordinating centre by telephone to receive the code number of the extended study drug bottles for a specific study participant.

Study treatment was stopped immediately if a patient developed symptoms or signs of a severe allergic reaction (grade 4),^15^ and treatment was completed with the second-line regimen (amoxicillin-clavulanic acid).^6^ Patients who were not able to tolerate the study drugs because of other adverse drug reactions and those who had an inadequate response to the study drugs as judged by the treating physician were also switched to amoxicillin-clavulanic acid. Amoxicillin-clavulanic acid was prescribed according to a weight-based dosage, as follows: bodyweight less than 60 kg: 1000 mg amoxicillin and 250 mg clavulanic acid three times daily, and bodyweight greater than 60 kg: 1500 mg amoxicillin and 375 mg clavulanic acid three times daily.^16^

### Outcomes and data management

The primary outcome of the study was culture-confirmed recurrent melioidosis, which was defined as the development of new symptoms and signs of infection in association with at least one culture from any site positive for *B pseudomallei* at any time between enrolment and study completion. Secondary endpoints were overall recurrent melioidosis (culture-confirmed recurrent melioidosis plus clinical recurrence), mortality, treatment failure, extension of treatment, and adverse drug reactions. The published protocol contains an error and states that mortality was a primary rather than a secondary outcome measure (appendix). Clinical recurrence was defined as the development of new symptoms and signs of infection that were consistent with melioidosis but in the absence of a positive *B pseudomallei* culture from any site. Treatment failure was defined as the clinical decision to change oral treatment as a result of inadequate response. Extension of treatment was defined as a decision by the treating physician to continue the study drug beyond 20 weeks. Adverse drug reactions were graded according to the National Cancer Institute Common Toxicity Criteria.^15^

### Genotyping and definition of relapse and re-infection

We compared *B pseudomallei* isolated on enrolment and at the recurrent episode using a combination of pulsed-field gel electrophoresis and multilocus sequence typing, as described previously.^6^, ^17^ Relapse and re-infection were defined on the basis of bacterial typing. Isolates from the same patient with an identical banding pattern on pulsed-field gel electrophoresis were considered to be from the same strain and these patients were classified as having had a relapse. Isolates from the same patient that differed by one or more bands were further examined with multilocus sequence typing.^6^, ^17^ Isolates from the same patient with a different sequence type were classified as representing re-infection, whereas those with an identical sequence type were classified as being a relapse.

### Statistical analysis

The trial was powered for non-inferiority at the completion of the study.^18^, ^19^ We chose a non-inferiority design rather than a superiority design because the intervention group (TMP-SMX plus placebo) received fewer antimicrobials than the control group (TMP-SMX plus doxycycline), and we hypothesised that the intervention regimen was not superior to the standard regimen (TMP-SMX plus doxycycline), but had equivalent efficacy. The expected incidence rate for the primary endpoint (culture-confirmed recurrent melioidosis) was based on the results of the most recent, previous randomised controlled trial.^7^ The non-inferiority margin was defined by the scientific committee as a hazard ratio for culture-confirmed recurrent melioidosis of 1·7. We calculated that 600 participants were needed to determine non-inferiority with a power of 80% at an alpha error of 5%.

All patients were analysed on the basis of their original allocation group (intention-to-treat analysis). Continuous variables are presented as median (IQR). IQRs are presented in terms of 25th and 75th percentiles. Categorical data are presented as number (%). We did survival analyses using the Kaplan-Meier method and Cox proportional hazard models. Time was measured from the day of study enrolment. For the primary analysis (intention-to-treat analysis) with culture-confirmed recurrent melioidosis as the failure outcome, participants were censored on the day of last follow-up or death due to other causes. We tested non-inferiority of the TMP-SMX plus placebo group by calculating the HR for the efficacy of TMP-SMX plus placebo over the TMP-SMX plus doxycycline, and compared the upper limit of the 95% CI to the non-inferiority margin. To accept the non-inferiority of TMP-SMX plus placebo to TMP-SMX plus doxycycline, the upper limit of the 95% CI needed to be equal to or less than 1·7. A one sided-test at an alpha error of 5% was also calculated for the non-inferiority margin. For the secondary analyses with treatment changes and treatment failure as the outcomes, participants were censored on the day of recurrent melioidosis or death due to other causes. We calculated the probabilities of event outcomes at each timepoint using the Kaplan-Meier method. For the Cox Proportional Hazard model, we assessed whether the hazard ratio was constant over time using Schoenfeld residuals. The analysis was repeated for the primary outcome in the per-protocol population, in which those who did not complete 20 weeks of study drug were excluded.

As recurrent melioidosis can be caused by either relapse or re-infection, we also assessed the efficacy of TMP-SMX for culture-confirmed relapse in a sensitivity analysis on the basis that we would not expect antimicrobial treatment to prevent re-infection after treatment has been completed. We did this using culture-confirmed relapse as a failure outcome and excluding patients who had no paired bacterial isolates available for genotyping from the first and recurrent episode and those with paired isolates and genotyping results that confirmed re-infection.

For safety analyses, we assessed the proportion of patients who had adverse drug reactions by treatment group. We compared proportions using the Fisher's exact test and continuous variables using the Mann-Whitney test. We used STATA (version 12.1) for all statistical analyses.

### Role of the funding source

The sponsors had no role in data collection, data analyses, data interpretation, or writing of the report. The corresponding author had full access to all the data in the study. All authors participated in the final discussion and approved the submission for publication.

## Results

**Figure 1:**
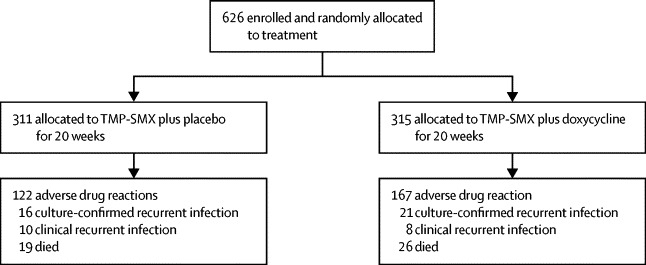
Trial profile TMP-SMX=trimethoprim-sulfamethoxazole.

Between Oct 24, 2005, and Feb 1, 2010, we randomly assigned 626 patients with culture-confirmed melioidosis to receive either oral TMP-SMX plus placebo (n=311), or oral TMP-SMX plus doxycycline (n=315; figure 1). Baseline characteristics were comparable between the two treatment groups (table 1). There were no missing data for baseline characteristics. Overall, 40 patients (6%) did not require parenteral antimicrobial treatment before enrolment, and 357 patients (57%) were deemed to need longer than 14 days of parenteral treatment before starting oral treatment. Most patients had a bodyweight between 40 kg and 60 kg and received 320 mg of TMP with 1600 mg of SMX twice daily (table 1).

**Table 1:** Baseline characteristics

		**TMP-SMX plus placebo group (n=311)**	**TMP-SMX plus doxycycline group (n=315)**
Study site			
	Sappasithiprasong Hospital, Ubon Ratchathani	178 (57%)	184 (58%)
	Srinagarind Hospital, Khon Kaen	47 (15%)	46 (15%)
	Udon Thani Hospital, Udon Thani	39 (13%)	38 (12%)
	Mahasarakam Hospital, Mahasarakam	24 (8%)	25 (8%)
	Khon Kaen Hospital, Khon Kaen	23 (7%)	22 (7%)
Men		198 (64%)	193 (61%)
Age in years		51 (41–61)	50 (40–59)
Underlying diseases			
	Diabetes mellitus	196 (63%)	217 (69%)
	Renal stones	19 (6%)	23 (7%)
	Chronic kidney disease	17 (5%)	14 (4%)
	Thalassaemia	12 (4%)	10 (3%)
	Other diseases*	15 (5%)	17 (5%)
Distribution of melioidosis†			
	Localised	129 (41%)	134 (43%)
	Multifocal	35 (11%)	51 (16%)
	Bacteraemic	99 (32%)	89 (28%)
	Disseminated	48 (15%)	41 (13%)
Organ involvement‡			
	Pneumonia	99 (32%)	109 (35%)
	Skin or subcutaneous abscess	93 (30%)	100 (32%)
	Splenic abscess	61 (20%)	56 (18%)
	Liver abscess	46 (15%)	44 (14%)
	Arthritis or osteomyelitis	24 (8%)	32 (10%)
	Urinary tract infection	22 (7%)	22 (7%)
	Lymphadenopathy	4 (1%)	9 (3%)
	Other§	12 (5%)	10 (4%)
Duration of parenteral antimicrobials before study drug			
	None	17 (5%)	23 (7%)
	1–14 days	113 (36%)	116 (37%)
	15–28 days	127 (41%)	129 (41%)
	≥29 days	54 (17%)	47 (15%)
Estimated glomerular filtration rate			
	≥60 mL/min per 1·73m^2^	188 (60%)	197 (63%)
	30–59 mL/min per 1·73m^2^	99 (32%)	89 (28%)
	15–29 mL/min per 1·73m^2^	24 (8%)	29 (9%)
Dosage of TMP-SMX received at enrolment			
	160/800 mg twice daily	25 (8%)	31 (10%)
	240/1200 mg twice daily	250 (80%)	241 (76%)
	320/1600 mg twice daily	36 (12%)	43 (14%)

Data are n (%) or median (IQR). TMP-SMX=trimethoprim-sulfamethoxazole.

* Included steroid intake (12 patients), cirrhosis (eight patients), haemoglobinopathy (seven patients), chronic liver disease (six patients), cancer (six patients), and immunosuppressive drug intake (four patients).

† Localised was defined as a single focus of infection and a negative blood culture result, multifocal as more than one contiguous focus of infection and a negative blood culture result, bacteraemic as a positive blood culture result plus a single or no identifiable focus of infection, and disseminated as a positive blood culture result plus more than one non-contiguous focus of infection.

‡ Organ involvement was defined as the presence of clinical features or clinical specimen taken from the organ that was culture positive for *Burkholderia pseudomallei*.

§ Included parotid abscess (five patients), mycotic aneurysm (four patients), central nervous system infection (three patients), prostatic abscess (two patients), eye infection (two patients), pericarditis (two patients), pancreatic abscess (one patient), sinusitis (one patient), cervicitis (one patient), and tubo-ovarian abscess (one patient).

Follow-up was completed in Feb 21, 2011, 1 year after we enrolled the last patient. 618 patients (99%) had at least one follow-up assessment. Median follow-up duration was 17 months (IQR 13–28) in the TMP-SMX plus placebo group and 19 months (16–31) in the TMP-SMX plus doxycycline group (p=0·06). Total duration of follow-up was 536 person-years in the TMP-SMX plus placebo group and 583 person-years in the TMP-SMX plus doxycycline group.

**Figure 2:**
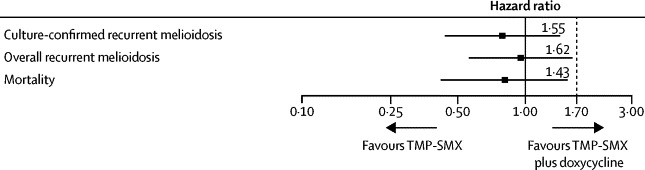
Non-inferiority of TMP-SMX relative to TMP-SMX plus doxycycline Datapoints are the point estimate of the hazard ratio (HR) between the trimethoprim-sulfamethoxazole (TMP-SMX) plus placebo group and TMP-SMX plus doxycycline group. Error bars are 95% CI. Clinical equivalence of TMP-SMX would be accepted if the upper bound of the 95% CI of the HR for culture-confirmed recurrent melioidosis (primary endpoint) was below the pre-defined non-inferiority margin (HR 1·7; dotted line).

**Figure 3:**
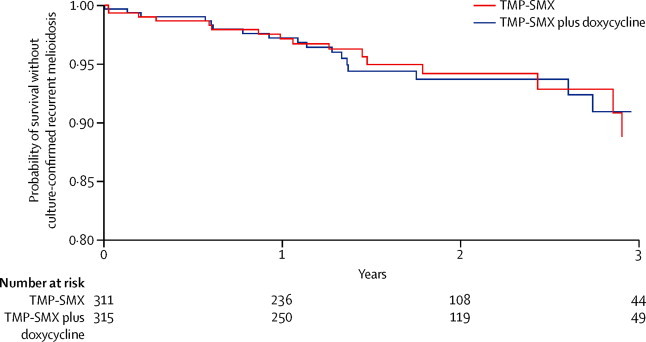
Kaplan-Meier curves of probability without culture-confirmed recurrent melioidosis TMP-SMX=trimethoprim-sulfamethoxazole.

We recorded no between-group difference in our primary analysis for culture-confirmed recurrent melioidosis (p=0·64; table 2). Non-inferiority of TMP-SMX plus placebo was shown because the upper bound of the 95% CI was below the pre-defined non-inferiority margin (one-sided p=0·01; figure 2). The probability of having culture-confirmed recurrent melioidosis within 1 year of enrolment was 3% and within 3 years of enrolment was 10% (figure 3). Of 37 culture-confirmed recurrent melioidosis cases, seven (19%) occurred during 20 weeks of oral treatment, 17 (46%) occurred during the first year of follow-up, four (11%) occurred between year 1 and year 2 of follow-up, and nine (24%) occurred after 2 years.

**Table 2:** Outcomes of the study

		**TMP-SMX plus placebo group (n=311)**	**TMP-SMX plus doxycycline group (n=315)**	**Hazard ratio (95% CI; p value)**
Recurrent melioidosis				
	Culture-confirmed	16 (5%)	21 (7%)	0·81 (0·42–1·55; p=0·64)
	Clinical	10 (3%)	8 (3%)	..
	Overall	26 (8%)	29 (9%)	0·95 (0·56–1·62; p=0·85)
Mortality				
	Due to recurrent melioidosis	8 (3%)	3 (1%)	..
	Due to other causes	11 (4%)	23 (7%)	..
	Overall	19 (6%)	26 (8%)	0·79 (0·44–1·43; p=0·44)
Discontinued study drug				
	Due to adverse event	37 (12%)	59 (19%)	0·61 (0·41–0·92; p=0·02)
	Due to treatment failure	6 (2%)	6 (2%)	0·92 (0·30–2·87; p=0·89)
Extended study drug		9/226 (4%)	12/218 (6%)	..

Data are n (%) or n/N (%), unless otherwise stated. TMP-SMX=trimethoprim-sulfamethoxazole.

Comparison of secondary endpoints in the two treatment groups is shown in table 2. The incidence of overall recurrent melioidosis and overall mortality was not different between the two treatment groups (table 2). Of 45 participants who died, 14 (31%) died during 20 weeks of oral treatment, 15 (33%) died during the first year of follow-up, six (13%) died between year 1 and year 2 of follow-up, and ten (22%) died after 2 years. Cause of death was culture-confirmed recurrent melioidosis (seven patients), clinical recurrent melioidosis (four patients), unknown causes (11 patients), and other diseases (23 patients). No deaths were attributed to an adverse reaction to the study drugs.

Overall, 516 (82%) patients received oral treatment for at least 12 weeks (including amoxicillin-clavulanic acid if patients needed switching) and 445 (71%) patients received oral treatment for at least 20 weeks. 37 (12%) of 311 patients given TMP-SMX plus placebo and 59 (19%) of 315 patients given TMP-SMX plus doxycycline switched treatment to amoxicillin-clavulanic acid because of adverse drug reactions. Patients given TMP-SMX plus placebo had about a 40% lower chance of switching to the second-line regimen due to adverse drug reactions than those given TMP-SMX plus doxycycline (HR 0·61; 95% CI 0·41–0·92; p=0·02). Six patients (2%) given TMP-SMX plus placebo and six patients (2%) given TMP-SMX plus doxycycline switched due to treatment failure (HR 0·92; 0·30–2·87; p=0·89). Analysis of Schoenfeld residuals showed that the HR for all outcomes were not variable over time with the exception of switching due to treatment failure (Schoenfeld test, p=0·05). Of all patients who completed 20 weeks of the study drug, nine (4%) of 226 patients in the TMP-SMX plus placebo group and 12 (6%) of 218 patients in the TMP-SMX plus doxycycline group needed an extension of treatment beyond 20 weeks (p=0·51).

The proportion of patients reporting adverse drug reactions was lower in the TMP-SMX plus placebo group than in the TMP-SMX plus doxycycline group (table 3). Common adverse drug reactions were allergic reactions and gastrointestinal disorders. Serious adverse events (grade 4) were reported in five patients (2%) given TMP-SMX plus placebo and eight patients (3%) given TMP-SMX plus doxycycline. These serious adverse events included Stevens-Johnson syndrome (three patients), severe hyponatraemia (<120 mmol/L; two patients), and severe hyperkalaemia (>7 mmol/L; one patient).

**Table 3:** Adverse drug reactions

		**TMP-SMX plus placebo group (n=311)**	**TMP-SMX plus doxycycline group (n=315)**
Allergic reactions			
	Rash	25 (8%)	48 (15%)
	Pruritus	8 (3%)	7 (2%)
	Photosensitivity	0	22 (7%)
	Stevens-Johnson syndrome	0	3 (1%)
Gastrointestinal disorders			
	Increased aspartate aminotransferase or alanine aminotransferase concentrations (>5 × ULN)	7 (2%)	9 (3%)
	Nausea	20 (6%)	45 (14%)
	Vomiting	21 (7%)	47 (15%)
	Anorexia	3 (1%)	5 (2%)
	Dyspepsia	5 (2%)	5 (2%)
Genitourinary disorder			
	Increased creatinine concentrations (≥ two times compared with baseline)	10 (3%)	9 (3%)
Haematological disorder			
	Anaemia (haemoglobin concentrations decreased ≥2 g/dL)	16 (5%)	20 (6%)
	Thrombocytopenia (<50 000 per mm^3^)	3 (1%)	7 (2%)
Metabolic disorder			
	Hyponatraemia (<130 mmol/L)	28 (9%)	19 (6%)
	Hyperkalaemia (>6 mmol/L)	13 (4%)	13 (4%)
	Hypokalaemia (<3 mmol/L)	6 (2%)	3 (1%)
Musculoskeletal disorders			
	Myalgia	2 (1%)	5 (2%)
Neurological disorders			
	Dizziness	2 (1%)	2 (1%)
Other adverse drug reactions*		7 (2%)	13 (4%)
Overall		122 (39%)	167 (53%)

Data are n (%). TMP-SMX=trimethoprim-sulfamethoxazole. ULN=upper limit of normal.

* Included nail change (three patients), peripheral oedema (three patients), generalised allergic reaction (two patients), headache (two patients), malaise (two patients), glossitis (two patients), alopecia (two patients), diarrhoea (one patient), insomnia (one patient), tinnitus (one patient), and hypoglycaemia (one patient).

We analysed 226 patients in the TMP-SMX plus placebo group and 218 patients in the TMP-SMX plus doxycycline group who completed 20 weeks of the study drug in a per-protocol analysis. Non-inferiority of TMP-SMX plus placebo for culture-confirmed recurrent melioidosis was also shown (HR 0·50, 95% CI 0·19–1·32).

We did bacterial genotyping for 29 (78%) of 37 patients with culture-confirmed recurrent melioidosis for whom paired isolates (from the primary and recurrent episode) were available. 14 recurrent cases (48%) were defined as relapse, and 15 recurrent cases (52%) were defined as re-infection. Median time to relapse (7 months, IQR 6–13) was shorter than median time to re-infection (29 months; 13–37; p=0·0009).

In a sensitivity analysis (n=603), we excluded 15 patients who developed genotype confirmed re-infection and eight patients who did not have paired isolates for genotyping (appendix). These results did not differ substantially from the main analysis, although the 95% CIs were wider as a consequence of the inclusion of fewer failure outcomes. Baseline characteristics were comparable between the two treatment groups (appendix). Of the 14 patients with culture-confirmed relapse, five were in the TMP-SMX plus placebo group (2% of 300 patients in this group included in the sensitivity analysis), and nine were in the TMP-SMX plus doxycycline group (3% of the 303 patients in the group; HR 0·58, 95% CI 0·19–1·73; p=0·33; appendix). The lower boundary of the 95% CI for the HR for culture-confirmed relapse was slightly greater than the non-inferiority margin (1·73 *vs* 1·70). In this analysis, 14 patients (5%) given TMP-SMX plus placebo and 24 patients (8%) given TMP-SMX plus doxycycline died during the study period (HR 0·61, 0·31–1·17; p=0·14; appendix). Patients given TMP-SMX plus placebo also had about a 40% lower chance of switching to the second-line regimen due to adverse drug reactions than did those given TMP-SMX plus doxycycline (HR 0·56, 0·3–0·85; p=0·01; appendix).

## Discussion

Our findings suggest that TMP-SMX is not inferior to TMP-SMX plus doxycycline as an oral treatment for melioidosis. Fewer patients given TMP-SMX plus placebo had adverse drug reactions or switched to the second-line regimen compared with those given TMP-SMX plus doxycycline. These findings provide the evidence for a change to the recommended oral regimen for melioidosis to TMP-SMX alone.

The probability of having culture-confirmed recurrent melioidosis within 1 year of enrolment into the study (3%) was lower than that seen in another trial (6%),^7^ in which the minimum duration of oral TMP-SMX and doxycycline was 12 weeks. Other factors that might explain the difference in recurrence include better drug compliance in this study resulting from pill counting and phone contact to improve follow-up, which were not done in the other trial.^7^ In this study, 82% of participants received oral treatment for at least 12 weeks compared with only 69% in the previous trial.^7^ Moreover, fewer patients in the TMP-SMX plus doxycycline group switched to the second-line drug regimen in this study compared with patients given the same treatment regimen in the previous trial (21% *vs* 25%).^7^ Fewer switches to the second-line drug regimens by the treating clinicians in this study might be associated with the finding of a retrospective study that showed a doubling in risk of relapse (HR 2·1) associated with second-line regimens (including amoxicillin-clavulanic acid) compared with TMP-SMX-based regimens.^6^

Findings from our bacterial genotyping analysis showed that half of recurrent infection was due to relapse, with the remainder being due to re-infection. This finding contrasts with the findings of a retrospective study of 921 patients with melioidosis over an 8 year period, in which 74% of recurrent infections (80 of 116) were due to relapse and 26% (30 of 116) were due to re-infection.^6^ One possible explanation is that in the retrospective study, 50% of 921 patients were initially treated with non TMP-SMX-based regimens which were associated with a higher rate of relapse. Use of genotyping to differentiate between relapse and re-infection in melioidosis is well described.^6^, ^20^ The chance of re-infection with *B pseudomallei* that belongs to the same genotype as that causing the primary infection is low because the genetic diversity of *B pseudomallei* strains in soil and water is high.^21^, ^22^ Furthermore, the chance that relapse is caused by an isolate that belongs to a different genotype to that of the isolate cultured during the primary infection would require mixed infection with two or more bacterial strains, the incidence of which is very low.^23^ Our findings indicate that patients with melioidosis who are starting oral treatment should receive education about the prevention of melioidosis re-infection, including avoiding direct contact with soil or environmental water, avoiding outdoor exposure to heavy rain or dust clouds, and not drinking untreated water.^24^ Such strategies should be practical in Thailand: findings from our case-control study showed that a proportion of participants wore boots while farming and drank boiled water.^24^ Further studies are needed to assess the effectiveness of preventive education given to populations at risk of re-infection, as well as those at risk for a first infective episode.

Although our study, to the best of our knowledge, is the largest randomised controlled trial to compare two drug regimens for the oral treatment of melioidosis and the first to use a non-inferiority design, it has several potential limitations. The first relates to the non-inferiority margin (HR 1·7), which was selected by the scientific committee on the basis of a balance between trial feasibility (completion within 4 years) and statistical robustness. Some investigators have suggested the adoption of a larger non-inferiority margin (HR 2·0), but we considered it unacceptable to adopt a new regimen that could actually have two times the risk of recurrent culture-confirmed melioidosis than the current standard. Some investigators have also suggested the adoption of a smaller non-inferiority margin (HR 1·5), but this would have required an 8 year study period with a sample size of 1200 patients, which we thought to be unfeasible. The scientific committee agreed that an upper boundary of the 95% CI of a HR that was equal to or less than 1·7 would be sufficient to change clinical practice to the use of TMP-SMX alone for oral treatment of melioidosis. Additional limitations were that 11 patients who died at home of unknown cause might have died from recurrent melioidosis, that eight (21%) of 37 paired *B pseudomallei* isolates were not available for genotyping, and that non-inferiority was not shown in a sensitivity analysis. We also acknowledge that CLSI do not provide criteria for the interpretation of disc diffusion and Etest assays for *B pseudomallei,*^11^ but these were used on the basis that they have been described previously,^12^, ^25^ are in common use in Thailand, and were the only feasible option to establish susceptibility in the five study sites. The estimated HR of discontinuation of the study drug due to treatment failure (HR 0·92; table 2) should be interpreted with caution because the Schoenfeld test provided weak evidence suggesting that the HR for this outcome might not be constant over time.

In this study, we also assessed the efficacy of TMP-SMX over TMP-SMX plus doxycycline for culture-confirmed relapse. The results from a sensitivity analysis were very similar to the main analysis, except that the lower bound of the 95% CI for the HR for culture-confirmed relapse was slightly greater than the non-inferiority margin. This finding is mainly because the study was not powered to assess the non-inferiority based on this outcome in the sensitivity analysis. Therefore, we would suggest that these potential limitations are unlikely to have affected the conclusions of the study.

Having established that TMP-SMX is preferable to TMP-SMX and doxycycline for the oral phase of melioidosis treatment (panel), the next challenge is to establish the optimum duration of this regimen.

Panel **Research in context**

**Systematic review**

We searched PubMed for randomised controlled trials that assessed oral antimicrobial treatment for melioidosis published in English between Jan 1, 1921, and Dec 31, 2012, using the following MeSH terms (“melioidosis” OR “pseudomallei”) AND “trial”. We searched bibliographies from selected studies for secondary references. We identified four randomised controlled trials (Rajchanuvong and colleagues,^26^ Chaowagul and colleagues,^27^ Chetchotisakd and colleagues,^28^ and Chaowagul and colleagues^7^). All four trials used culture-confirmed recurrent melioidosis as the primary outcome. The trial done by Rajchanuvong et al^26^ compared amoxicillin-clavulanic acid with a combination of TMP-SMX, doxycycline, and chloramphenicol. Their findings suggested that amoxicillin-clavulanic acid was less effective, and they recommended that amoxicillin-clavulanic acid should be used as a second-line regimen. Chaowagul and colleagues^27^ compared doxycycline alone with a combination of TMP-SMX, doxycycline, and chloramphenicol, and showed that doxycycline alone was not effective for the treatment of melioidosis. Chetchotisakd and colleagues^28^ compared oral ciprofloxacin plus azithromycin with TMP-SMX plus doxycycline and showed that ciprofloxacin plus azithromycin was not effective for the treatment of melioidosis. The trial published by Chaowagul and colleagues^27^ in 2005 compared TMP-SMX plus doxycycline with a combination of TMP-SMX, doxycycline, and chloramphenicol. TMP-SMX plus doxycycline was not different from a combination of TMP-SMX, doxycycline, and chloramphenicol, and TMP-SMX plus doxycycline was recommended as the standard for oral treatment on the basis of fewer side-effects.

**Interpretation**

To our knowledge, our trial is the first to compare TMP-SMX alone with TMP-SMX plus doxycycline as oral antimicrobial treatment for melioidosis, and the first melioidosis trial to use a non-inferiority design. Both regimens (TMP-SMX alone and TMP-SMX plus doxycycline) are used in clinical practice. Our trial shows that TMP-SMX alone is not inferior to TMP-SMX plus doxycycline, and we propose that TMP-SMX alone should be used on the basis of safety and patient tolerance.
