# Supplementary material for: Trimethoprim-sulfamethoxazole versus trimethoprim-sulfamethoxazole plus doxycycline as oral eradicative treatment for melioidosis (MERTH): a multicentre, double-blind, non-inferiority, randomised controlled trial
Source: Lancet. 2014 Mar 1;383(9919):807–14. doi: 10.1016/S0140-6736(13)61951-0 (PMC3939931; doi:10.1016/S0140-6736(13)61951-0)
Supplement: Supplementary appendix [file mmc1.pdf]

THE LANCET

Supplementary appendix

This appendix formed part of the original submission and has been peer reviewed. We post it as supplied by the authors.

Supplement to: Chetchotisakd P, Chierakul W, Chaowagul W, et al. Trimethoprim-sulfamethoxazole versus trimethoprim-sulfamethoxazole plus doxycycline as oral eradicative treatment for melioidosis (MERTH): a multicentre, double-blind, non-inferiority, randomised controlled trial. *Lancet* 2013; published online Nov 25. http://dx.doi.org/10.1016/S0140-6736(13)61951-0.

Supplementary information:

A sensitivity analysis of culture-confirmed relapse in 603 patients, which excludes 15 patients who developed genotype-confirmed re-infection and 8 patients who did not have paired isolates for genotyping.

Table S1: Baseline characteristics

Characteristics	TMP-SMX plus placebo group (n=300)	TMP-SMX plus doxycycline group (n=303)
Study site		
Sappasithiprasong Hospital, Ubon Ratchathani	169 (56%)	174 (57%)
Srinagarind Hospital, Khon Kaen	47 (16%)	45 (15%)
Udon Thani Hospital, Udon Thani	38 (13%)	37 (12%)
Mahasarakam Hospital, Mahasarakam	24 (8%)	25 (8%)
Khon Kaen Hospital, Khon Kaen	22 (7%)	22 (7%)
Sex, men	191 (64%)	188 (62%)
Age, years	51 (41-61)	50 (41-59)
Underlying diseases		
Diabetes mellitus	190 (63%)	208 (69%)
Renal stones	17 (6%)	23 (7%)
Chronic kidney disease	14 (5%)	14 (5%)
Thalassemia	12 (4%)	9 (3%)
Other diseases*	15 (5%)	15 (5%)
Distribution of melioidosis**		
Localized	126 (42%)	132 (44%)
Multifocal	35 (12%)	49 (16%)
Bacteraemic	93 (31%)	85 (28%)
Disseminated	46 (15%)	37 (12%)
Organ involvement ***		
Pneumonia	93 (31%)	106 (35%)
Skin or subcutaneous abscess	93 (31%)	95 (31%)

Splenic abscess	59 (20%)	52 (17%)
Liver abscess	44 (15%)	41 (14%)
Arthritis or osteomyelitis	24 (8%)	30 (10%)
Urinary tract infection	20 (7%)	22 (7%)
Lymphadenopathy	4 (1%)	9 (3%)
Other ****	12 (5%)	10 (4%)
Duration of parenteral antimicrobials prior to study drug		
None	17 (6%)	23 (7%)
1-14 days	108 (36%)	111 (37%)
15-28 days	121 (40%)	123 (41%)
≥ 29 days	54 (18%)	46 (15%)
Baseline estimated GFR		
≥ 60 mL/min per 1.73m^2	184 (61%)	188 (62%)
30 – 59 mL/min per 1.73m^2	95 (32%)	86 (28%)
15 – 29 mL/min per 1.73m^2	21 (7%)	29 (10%)
Dosage of TMP-SMX received at enrollment		
160/800 mg twice daily	24 (8%)	30 (10%)
240/1200 mg twice daily	241 (80%)	232 (77%)
320/1600 mg twice daily	35 (12%)	41 (13%)

Data are n (%) or median (interquartile range).

* Included steroid intake (11), cirrhosis (8), hemoglobinopathy (6), chronic liver disease (6), cancer (6), and immunosuppressive drug intake (4)

** Localized was defined as a single focus of infection and a negative blood culture result, multifocal as >1 contiguous focus of infection and a negative blood culture result, bacteraemic as a positive blood culture result plus a single or no identifiable focus of infection, and disseminated as a positive blood culture result plus >1 noncontiguous focus of infection

*** Organ involvement was defined as the presence of clinical features and/or clinical specimen taken from the organ that was culture positive for *B. pseudomallei*

**** Included parotid abscess [5], mycotic aneurysm [4], central nervous system infection [3], prostatic abscess [2], eye infection [2], pericarditis [2], pancreatic abscess [1], sinusitis [1], cervicitis [1] and tubo-ovarian abscess [1]

Table S2. Outcomes of the study

	TMP-SMX plus placebo group (n=300)	TMP-SMX plus doxycycline group (n=303)	Hazard ratio (95% CI)
Recurrent melioidosis			
Culture-confirmed relapse	5 (2%)	9 (3%)	0.58 (0.19-1.73)
Mortality			
Due to culture-confirmed relapse	2 (1%)	1 (1%)	-
Due to other causes	12 (4%)	23 (7%)	-
Overall	14 (5%)	24 (8%)	0.61 (0.31-1.17)
Discontinued study drug			
Due to adverse event	33 (11%)	57 (19%)	0.56 (0.36-0.85)
Due to treatment failure	6 (2%)	6 (2%)	0.75 (0.23-2.46)
Extended study drug	8 / 221 (4%)	12 / 208 (6%)	-

Data are n (%). CI=confidence interval

MERTH study group

Ploenchan Chetchotisakd, Siriluck Anunnatsiri, Piroon Mootsikapun, Bandit Thinkhamrop, Surasakdi Wongrattanacheewin (Khon Kaen University, Thailand), Vipada Chaowagul, Prapit Teparrugkul (Sappasithiprasong Hospital, Thailand), Kriangsak Phimda, Sunchai Phiphitaporn, Sunee Chueasuwanchai, Pornpan Suntornsut (Udon Thani Hospital, Thailand), Sakesan Chaisuksan, Wattanachai Susaenrat (Khon Kaen Hospital, Thailand), Jiraporn Pilaikul, Chalongchai Toondee (Mahasarakam Hospital, Thailand), Pitchayanant Ariyaprasert, Narisara Chantratita, Wirongrong Chierakul, Nicholas P Day, Atchriya Hemachandra, Direk Limmathurotsakul, Sharon J Peacock, Janjira Thaipadungpanit, Vanaporn Wuthiekanun, Prayoon Yuentrakul (Faculty of Tropical Medicine).

Study Protocol for Registration

1) Project Title:

A Comparison of Doxycycline plus Trimethoprim-Sulphamethoxazole versus Trimethoprim-Sulphamethoxazole as Maintenance Therapy for Melioidosis

2) Investigators:

Principal investigator: Dr Ploenchan Chetchotsakd [1]

Co-investigators: Professor Wipada Chaowagul[2]
Dr Wattanachai Susaengrat[3]
Dr Siriluck Anunnatsiri[1]
Dr Piroon Mootsikapun[1]
Dr Wirongrong Chierakul[4]
Dr Kriangsak Pimda[5]
Dr Sakesan Chaisuksan[3]
Dr Sunchai Piphitaporn[5]
Dr Sunee Chueasuwanchai[4]
Dr Chalongchai Toondee[6]
Dr Jiraporn Pilaikul[6]
Dr Direk Limmathurotsakul[4]
Prof Nicholas Day[4]
Prof Sharon Peacock[4]

1. Faculty of Medicine, Srinagarind Hospital, Khon Kaen University, Khon Kaen, Thailand
2. Medical Department, Sappasithiprasong Hospital, Ubon Ratchathani, Thailand
3. Medical Department, Khon Kaen Hospital, Khon Kaen, Thailand
4. Department of Clinical Tropical Medicine, Faculty of Tropical Medicine, Mahidol University, 420/6 Rajvithi Road, Bangkok, Thailand
5. Medical Department, Udon Thani General Hospital, Udon Thani, Thailand
6. Medical Department, Mahasarakam Hospital, Mahasarakam, Thailand

3) Summary

We propose a prospective, non-inferiority, double-blind, randomised, placebo-control trial of a three-drug combination (doxycycline-trimethoprim (TMP)-sulfamethoxazole (SMX)) versus two-drug combination (TMP-SMX) for the oral maintenance treatment of melioidosis.

4) Background

Adults presenting with melioidosis are treated with parenteral therapy (usually ceftazidime) for two weeks after presentation, followed by oral therapy for a further 12 to 20 weeks. This prolonged course is required to reduce the rate of relapse. In our experience, relapse occurs in approximately 10% of those with primary infection who receive 20 weeks of antibiotic treatment, rising to almost one quarter in those treated for 8 weeks (1). The conventional oral 'maintenance' treatment used is a four-drug combination of chloramphenicol, doxycycline, TMP, and SMX. However, this is associated with poor compliance, most likely as a combined result of long treatment duration with a complicated regimen and drug side effects. Poor compliance has been shown to be the most significant risk factor for subsequent relapse (relative risk 4.9, 95% CI 1.2-20.3) (2). It is crucial, therefore, to determine whether more simplified oral treatment regimens are of equal efficacy.

Several previous randomized controlled trials have been conducted. Comparison of the conventional four-drugs regimen with amoxicillin-clavulanic acid (co-amoxiclav) demonstrated that co-amoxiclav was better tolerated and could be administered to children and pregnant women, but was considerably more expensive and when used in short course (less than 12 weeks) was less effective (3). Comparison of ciprofloxacin plus azithromycin with cotrimoxazole plus doxycycline regimen was associated with 22% vs. 3% relapse rate (4). Four drugs versus doxycycline alone led to a 20% relapse rate in the doxycycline group (5). We have recently completed a prospective, randomized comparison of a three-drug regimen (without chloramphenicol) with conventional treatment. Both groups had no different in relapse rate and mortality rate, and the three

drug regimen was associated with fewer side effects (6). This three-drug combination regimen has also been reported to have been used in general practice (4). However, about 20% of all patients had to switch to other regimens of treatment because of drug side effects, most common of which was related to doxycycline (skin rashes, facial erythema, photosensitivity and vomiting).

Given the side effects caused by doxycycline, it seems rational to now study the efficacy of three drugs (doxycycline, TMP, and SMX) versus two drugs (TMP-SMX). This also offers a simple regimen for the patient. Retrospective analysis of treatment data in Australia has indicated that TMP-SMX treatment for eradication after the initial intensive phase was associated with a relapse rate of less than 2% (7,8). Furthermore, *in vitro* data provides support for the use of two rather than four drugs, since four drugs (conventional treatment) were found to be bacteriostatic and mutually antagonistic, while the combination of TMP and SMX was synergistic (9). One potential problem associated with the use of TMP-SMX is the difficulty in determining *in vitro* susceptibility using disc testing. This can be overcome by using the E-test which is reliable in evaluating antimicrobial susceptibility to TMP-SMX (10,11). E-test will be our standard for susceptibility testing, and susceptibility of a given isolate to TMP-SMX will be confirmed prior to entry into the study.

5) Objective

To evaluate the efficacy, effectiveness and compliance of TMP-SMX compared with doxycycline, TMP, and SMX in the oral maintenance phase treatment of melioidosis

6) Place and period

Sappasithiprasong Hospital, Ubon Ratchathani, Udon Thani Hospital, Udon Thani, Srinagarind Hospital and Khon Kaen General Hospital in Khon Kaen, Mahasarakam Hospital, Mahasarakam in north eastern Thailand between October 2005 and October 2009.

7) Materials and Methods

For the randomized controlled study, the study population will include all adult patients who have culture-confirmed melioidosis infection.

7.1 Sample

7.1.1 Inclusion criteria

1. Culture-confirmed melioidosis.
2. Satisfactory completion of intravenous therapy and able to take oral medication.
3. Patients with mild localized disease who are not considered to require intravenous treatment by their primary physician are eligible if they agree to return for follow up.
5. Age > 14 years.
6. High likelihood of completing at least 6 months follow up.
7. Willingness to participate in the study and written, informed consent obtained from the patient.

7.1.2 Exclusion criteria (any one of the following)

1. Pregnancy or breast feeding.
2. Contraindications to doxycycline: severe hepatic impairment (AST, ALT ≥5times of upper limit of normal)
3. Contraindications to TMP-SMX: G6PD, renal impairment (creatinine clearance <15ml/min
4. History of hypersensitivity to doxycycline, TMP or SMX.
5. Infecting isolate is resistant to TMP-SMX by E-test.
6. Previous melioidosis with disease free of less than 2 years

7.2 Methods

7.2.1 Study Design

Prospective, double-blind, non-inferiority, randomised, placebo controlled trial

Patients will be identified from examination of daily admission logs, ward rounds of medical, respiratory care and intensive care wards, and discussion with treating clinicians. Adult patients with culture confirmed melioidosis who have been successfully treated with intravenous antibiotics will be randomized into the trial after consent has been gained. Patients infected with an isolate resistant to TMP-SMX will not be included into the study and will be treated with co-amoxiclav. Patients who do not fulfill admission criteria for other reasons will be assessed, and the treatment choice guided by reason for study exclusion.

7.2.2 Baseline record

A full history, physical examination, laboratory investigations (including culture confirmation of diagnosis, chest x-ray and ultrasonography), and acute phase treatment regimen will be recorded by the study team.

7.2.3 Treatment

The patients will be randomized into two groups. The randomization will be done in advance by blocks of 10 and stratified by study site. The treatment drugs will be pre-packed in the same bottles and labeled with the study code. The study drugs include either of the combinations below:

three drugs:

Co-trimoxazole (10 mg TMP + 50 mg SMX/kg/d): 3 adult tablets (80mg TMP/tab), twice daily. The dose of co-trimoxazole will be reduced to 2 tablets, twice daily in case of patients with creatinine clearance less than 30 ml/min or weigh less than 40 kg, and increased to 4 tablets, twice daily in case of patients weigh more than 60 kg.

Doxycycline (200 mg/day): 1 tablet twice daily

two drugs:

Co-trimoxazole (10 mg TMP + 50 mg SMX/kg/d): 3 adult tablets (80mg TMP/tab), twice daily. The dose of co-trimoxazole will be reduced to 2 tablets, twice daily in case of patients with creatinine clearance less than 30 ml/min or weigh less than 40 kg, and increased to 4 tablets, twice daily in case of patients weigh more than 60 kg.

Placebo (identical tablet as doxycycline): 1 tablet twice daily

The duration of treatment will be minimum at 20 weeks. The maximum duration will be decided by the attending clinician for each individual patient, according to clinical progress.

7.2.4 Other investigations and patient monitoring during study

At each visit, symptoms and signs will be recorded; a complete blood count and standard blood chemistry will be performed during treatment and as clinical indicated after completed treatment. Appropriate cultures, chest radiography, and abdominal ultrasonography will be repeated if they are abnormal previously. Compliance with the drug therapy will be checked by interview and pill counting.

The patients will be examined as outpatients by one of the study team, initially at week 4, 12, and 20 of oral treatment, every 4 months for 1 year after completion of treatment, and annually thereafter to the end of the study. The patients will be advised to present between appointments if they develop symptoms suggestive of relapse.

7.2.5 Discontinuation criteria

All of the therapeutic drugs will be stopped immediately if the patient develops symptoms or signs of severe allergic reaction, and treatment completed using co-amoxiclav. Patients who have clinical features consistent with treatment failure will be switched to co-amoxiclav.

7.3 Outcome measurement

The primary outcome measures will be culture-confirmed recurrent melioidosis. This is defined as clinical features of melioidosis after initial improvement, in association with cultures from any site positive for *Burkholderia pseudomallei.*

Secondary outcome measures will include the following:

1. Mortality
2. Clinical recurrence; recurrent clinical features of melioidosis treated as such but not confirmed by positive culture.

3. Treatment failure: clinical decision to change treatment according to inadequate response to therapy

4. Adverse drug reactions, including drug allergy

5. Drug compliance: based on interview and pill counting

7.4 Statistical methods

7.4.1 Sample size

We aim to study the indifference of relapse rate at hazard ratio 1.7. With 95% confidence and 80% power, 600 culture-proven melioidosis cases are needed.

```
. artsurv, method(1) edf0(0.9 0.83,1 3) hr(1,1.7) ng(2) np(3) recrt(2 0,1)
lg(1 2) ldf(.1 .2 .3, 1 2 3; .1 .2 .3, 1 2 3) distant(0) detail(1) onesided(1)
ni(1) tunit(1) alpha(0.05) power(0.8)

ART - ANALYSIS OF RESOURCES FOR TRIALS        (version 1.0.4, 13 January 2005)
-------------------------------------------------------------------------------
A sample size program by Abdel Babiker, Patrick Royston & Friederike Barthel,
MRC Clinical Trials Unit, London NW1 2DA, UK.
-------------------------------------------------------------------------------
Type of trial                              Noninferiority - time-to-event
outcome
Statistical test assumed                   Unweighted logrank test (local)
Number of groups                           2
Allocation ratio                           Equal group sizes

Total number of periods                    3
Length of each period                      One year

Survival probs per period (group 1)        0.900 0.864 0.830
Survival probs per period (group 2)        0.836 0.780 0.729
Number of recruitment periods              2
Number of follow-up periods                1
Method of accrual                          Uniform
Recruitment period-weights                 1 1 0

Hazard ratios as entered (groups 1,2)      1, 1.7
Hazard ratios per period (group 1)         1.000 1.000 1.000
Hazard ratios per period (group 2)         1.700 1.700 1.700
Alpha                                      0.050 (one-sided)
Power (designed)                           0.800

Total sample size (calculated)             593
Expected total number of events            97
-------------------------------------------------------------------------------
```

7.4.2 Analysis plan

All patients will be analysed based on their original allocation group (intention-to-treat analysis). Cox proportional hazard models will be used. Time will be measured from the day of study enrollment. For the primary analysis with culture-confirmed recurrent melioidosis as the failure outcome, participants will be censored on the day of last follow-up or death due to other causes. To accept the non-inferiority of TMP-SMX/placebo to TMP-SMX/doxycycline, the upper limit of the 95%CI needs to be equal or less than 1.7. For the secondary analysis, Cox proportional hazard models, Fisher's exact tests and Mann-Whitney tests will be used. The analyses will be performed using STATA version 12.1 (StataCorp, College Station, TX).

8) Ethical considerations

8.1 Safety

All therapeutic drugs in this study are generally used in the treatment of melioidosis, and have been proved for their safety. Doxycycline may cause GI irritation, nausea, vomiting, and photosensitivity. GI irritation can be prevented by taking drug with foods. Placebo used in the study has no therapeutic effects, so the use of it is safe. There will be no extra blood taken for the purpose of the study. All blood taken and tests will be performed according to patient clinical management. We will report to the Ethical Committee, Ministry of Public Health immediately if any serious adverse effects occur due to the study drugs.

8.2 Informed Consent

The information concerning the study will be given in detail by a study nurse. The patients will be enrolled after she/he or their relatives agree and sign the consent form. A copy of the proposed consent form is attached.

The participants or their relatives can withdraw from the study at any time for any reason. After withdrawal, the study drugs will be ceased and conventional therapeutic regimen will be given at standard doses. Patient information will be kept confidentially by the

study team. Only the outcome of the study will be published, not any individual patient information.

8.3 Ethical clearance

This proposed study was approved by The Ethical Clearance from Ministry of Public Health, Royal Government of Thailand, Faculty of Medicine, Khon Kaen University, and The Oxford Tropical Research Ethics Committee (OXTREC), United Kingdom.

9) Funding

This study is collaboration between the Wellcome Trust-Mahidol University, Oxford University Tropical Medicine Research Programme, and Sappasithiprasong Hospital, Ubon Ratchathani, Udon Thani Hospital, Udon Thani, Mahasarakam Hospital, Mahasarakam, Srinagarind Hospital, Khon Kaen University and Khon Kaen Hospital, Khon Kaen, Thailand. All therapeutic drugs in this study are donated by Siam Pharmaceutical Company, Bangkok, Thailand.

10) References

1. White NJ. Melioidosis. Lancet 2003;361(9370):1715-22.
2. Chaowagul W, Suputtamongkol Y, Dance DA, Rajchanuvong A, Pattara-arechachai J, White NJ. Relapse in melioidosis: incidence and risk factors. J.Infect.Dis. 1993;168(5):1181-5.
3. Rajchanuvong A, Chaowagul W, Suputtamongkol Y, Smith MD, Dance DA, White NJ. A prospective comparison of co-amoxiclav and the combination of chloramphenicol, doxycycline, and co-trimoxazole for the oral maintenance treatment of melioidosis. Trans.R.Soc.Trop.Med.Hyg. 1995;89(5):546-9.
4. Chetchotisakd P, Chaowagul W, Mootsikapun P, Budhsarawong D, Thinkamrop B. Maintenance therapy of melioidosis with ciprofloxacin plus azithromycin compared with cotrimoxazole plus doxycycline. Am.J.Trop.Med.Hyg. 2001;64(1-2):24-7.
5. Chaowagul W, Simpson AJ, Suputtamongkol Y, Smith MD, Angus BJ, White NJ. A comparison of chloramphenicol, trimethoprim-sulfamethoxazole, and

doxycycline with doxycycline alone as maintenance therapy for melioidosis [In Process Citation]. Clin Infect Dis 1999;29(2):375-80.

6. Chaowagul W, Chierakul W, Simpson AJ, et al. Open-label randomized trial of oral trimethoprim-sulfamethoxazole, doxycycline, and chloramphenicol compared with trimethoprim-sulfamethoxazole and doxycycline for maintenance therapy of melioidosis. Antimicrob Agents Chemother. 2005 Oct;49(10):4020-5.
7. Currie BJ, Fisher DA, Howard DM, Burrow JN, Lo D, Selva-Nayagam S et al. Endemic melioidosis in tropical northern Australia: a 10-year prospective study and review of the literature. Clin.Infect.Dis. 2000;31(4):981-6.
8. Currie BJ, Fisher DA, Anstey NM, Jacups SP. Melioidosis: acute and chronic disease, relapse and re-activation. Trans.R.Soc.Trop.Med.Hyg. 2000;94(3):301-4.
9. Dance DA, Wuthiekanun V, Chaowagul W, White NJ. Interactions in vitro between agents used to treat melioidosis. J.Antimicrob.Chemother. 1989;24(3):311-6.
10. Piliouras P, Ulett GC, Ashhurst-Smith C, Hirst RG, Norton RE. A comparison of antibiotic susceptibility testing methods for cotrimoxazole with Burkholderia pseudomallei. Int.J.Antimicrob.Agents 2002;19(5):427-9.
11. Wuthiekanun V, Cheng AC, Chierakul W, et al. Trimethoprim/sulfamethoxazole resistance in clinical isolates of *Burkholderia pseudomallei.* J Antimicrob Chemother. 2005 Jun;55(6):1029-31.

INFORMATION SHEET

A comparison of doxycycline plus trimethoprim-sulphamethoxazole versus trimethoprim-sulphamethoxazole as maintenance therapy for melioidosis

You/your relative are being asked to join a research study of patients who have been diagnosed as having the infection called melioidosis. This is a collaborative project between Sappasithiprasong Hospital, Udon Thani General Hospital, Mahasarakam Hospital, Srinagarind Hospital and the Faculty of Tropical Medicine, Mahidol University. We would like to study if treatment with one type of pill (co-trimoxazole (trimethoprim-sulphamethoxazole)) alone is as effective as using two types of pill (co-trimoxazole plus doxycyline) for preventing a return of melioidosis.

You/your relative will be asked to give consent to our staff to collect data from you/your relative and medical records.

What is the purpose of the study?

The reason we wish to do this is because the oral treatment of melioidosis is usually longer than 3 months and involves taking a large number of tablets each day. Patients who take these drugs might experience side effects, and such problems may result in medicines being stopped or not taken correctly. We want to try and avoid patients stopping treatment early since this is the most important risk factor for relapse (return) of infection. Co-trimoxazole with doxycyline is currently the standard oral treatment. Co-trimoxazole alone has been used for treatment in patients with melioidosis in Australia and has been reported to be safe and effective, but this has not been assessed in a treatment trial.

Why have I been chosen?

You have been asked to join this research study because you have a definite diagnosis of melioidosis.

Do I have to take part?

It is up to you/your relative to decide whether or not to take part. If you/your relative do decide to take part you will be given this information sheet to keep and be asked to sign a consent form.

What will happen to me if I take part?

If you/your relative are willing to participate, you will be randomly assigned to receive either co-trimoxazole treatment plus a placebo (a tablet without pharmacological activity) OR co-trimoxazole plus doxycyline. In both cases you will be given two types of pills to take, but in the first case one of the pills will be a placebo (inactive)

You/your relative will be asked to come back to outpatient clinic for regular follow up. Samples of urine, sputum (if present), body fluid or pus (if present) may also be collected for culture; these are normally taken when investigating a patient with suspected return of melioidosis. These tests and investigations are part of the normal care of melioidosis patients, and no extra blood samples or investigations will be done because you are enrolled in the study.

What are the possible risks or side effects of the study drugs and procedures?

Doxycycline is a widely used antibiotic for the treatment of many infections. It has also been one of the recommended drugs for melioidosis treatment for more than 20 years. It is considered a safe drug. Possible adverse reactions are nausea, vomiting, rashes following exposure to sunlight (called photosensitivity) and discolouration of bones and teeth when used in children.

TMP and SMX are also widely used and safe. Possible side effects of the drugs include photosensitivity. These drugs cross the placenta and are distributed into breast milk. These may interfere with folic acid metabolism of the baby. Severe side effects such as

bad skin rashes, liver damage, and serious blood problems are rare. The drugs are convenient to take since they are combined in a single tablet.

The placebo pill used in this study is identical in appearance to doxycycline, but contain no active drug.

The volume of blood taken will not do any harm to you. The procedure used to take blood samples is the one routinely used. This can lead to bruising or inflammation of the vein, but will be performed by experienced, well-trained doctors or nurses to minimize this side effect. The organizing study team, which is from Khon Kaen University, will take full responsibility for any harm and complications which may be caused by the study procedures, without any cost to you/your relative.

What are the possible benefits of taking part?

You/your relative will not get any payment directly from the study. The drugs will be supplied by the study team without any charge to you/your relative. We hope that this study will give us information to help improve the treatment of the future patients.

Who will pay for travelling cost if I will be asked for follow up?

You/your relative will be asked to come back for follow up at the hospital every 4-8 weeks after discharge. The study team will pay between 100-250 baht for your travel (depending on the actual cost) for each follow up visit.

What if something goes wrong?

You/your relative have the right to ask questions at any time. You/your relative can withdraw from the study at any time and for any reason, and this will have no effect on the treatment you/your relative receive.

If any complication occurs as a result of the study, the study team (and ultimately Khon Kaen University) will be responsible and any extra treatment needed will be provided without any cost. There are no extra costs for you/your relative if you/your relative

decide to participate in this study. Any additional costs such as travel to outpatients for repeat tests will be compensated for.
We will report to the Ethical Committee, Ministry of Public Health immediately if any adverse effects happen due to the study procedures.

Confidentiality

Patient information will be kept confidential by the study team. Only the outcome of the study will be published, not any individual patient information.

Who has reviewed the study?

This study has been reviewed and approved by the ethics committee of The Ministry of Public Health, Royal Government of Thailand, Khon Kaen University, Khon Kaen, Thailand, and The Oxford Tropical Research Ethics Committee (OXTREC), United Kingdom.

Name of 24 hours contacts in case of any problems or questions:

Srinagarind Hospital

1. Dr.Ploenchan Chetchotsakd, Department of Medicine, Srinagarind Hospital. Tel: 043 363664 or Mobile phone: 01 5925907
2. Dr Piroon Mootsikapun, Department of Medicine, Srinagarind Hospital. Tel: 043 363664 or Mobile phone: 01 8721858
3. Dr Siriluck Anunnatsiri, , Department of Medicine, Srinagarind Hospital. Tel: 043 363664 or Mobile phone: 01 0511995

Sappasithiprasong Hospital

1. Dr Direk Limmathurotsakul: Melioidosis Laboratory, Sappasithiprasong Hospital. Tel: 045 246112, Mobile phone: 09 7543784
2. Dr Atchriya Hemachandra: Melioidosis Laboratory, Sappasithiprasong Hospital. Tel: 045 246112, Mobile phone: 06 5887665

3. Prof Wipada Chaowagul: Medical Department, Sappasithiprasong Hospital. Tel: 045 246112, Mobile phone: 01 8765372
4. Dr Wirongrong Chierakul: Melioidosis Laboratory, Sappasithiprasong Hospital. Tel: 045 246112 or Faculty of Tropical Medicine, Mahidol University: 02 3541395 or Mobile phone: 09 1058571

Khon Kaen Hospital

1. Dr Wattanachai Susaengrat, Medical Department, Khon Kaen Hospital. Tel: 043 236789 Ext 1174 or Mobile phone: 01 5444860
2. Dr Sakesan Chaisuksan, Medical Department, Khon Kaen Hospital. Tel: 043 236789 Ext 1174 or Mobile phone: 01 5440233

Udon Thani Hospital

1. Dr.Sunee Chueasuwanchai, Wellcome Laboratory, Udon Thani General Hospital. Tel: 042 347733 or Mobile phone: 01 8622202
2. Dr.Kriangsak Pimda, Medical Department, Udon Thani General Hospital. Tel: 042 244272 (Ext. 7162) or Mobile phone: 01 8717573
3. Dr Sunchai Piphitaporn, Medical Department, Udon Thani General Hospital. Tel: 042 244272 (Ext. 7162) or Mobile phone: 01 3205186
4. Dr Wirongrong Chierakul: Leptospirosis Laboratory, Udon Thani General Hospital. Tel: 042 347733 or Faculty of Tropical Medicine, Mahidol University: 02 3541395 or Mobile phone: 09 1058571

Mahasarakam Hospital

1. Dr Chalongchai Toondee, Mahasarakam Hospital Tel: 043 740937-9 or Mobile phone: 09 4229030
2. Dr Jiraporn Pilaikul, Mahasarakam Hospital Tel: 043 740937-9 or Mobile phone: 01 6614353

CONSENT FORM

A Comparison of Doxycycline, Trimethoprim-Sulphamethoxazole with Trimethoprim-Sulphamethoxazole Alone as Maintenance Therapy for Melioidosis

Date..... Study no.....

I have been given an information sheet on this research study and have discussed the study with _____

(name of doctor or nurse)

☐ I understand the purpose of the study.

☐ I understand why I have been chosen

☐ I understand that I do not have to take part of the study

☐ I am aware of the procedures involved in this study, including:

- Administration of co-trimoxazole with doxycycline or placebo for at least 20 weeks
- Blood samples and other investigations may be taken at each follow-up visit depending on my clinical condition
- Follow up visit to the hospital every one to four months, depending on my clinical progress, for 2 years. The compensation and travelling cost will be paid by the investigator for 100-250 Baht each visit.

☐ These procedures have been explained to me, including the possible risks, any discomfort involved, and length of time and the frequency with which the procedures will be performed. I have understood and am satisfied with the explanations that I have been given. I have been provided with a written information sheet.

☐ I understand that my involvement in this research project may not be of any direct benefit to me.

☐ I have the right to withdraw from the study at any time and for any reason. There will be no effect on my management should I decide to withdraw from the study.

☐ I understand that the researchers will take the responsibility for any harm or complications which may happen from the study procedures without any cost to me.

 My information will be kept confidential by the researcher. Only the outcome of the study can be presented or published, not my individual information.

I can contact the researchers at any time in case of any questions or problems:

Srinagarind Hospital

1. Dr.Ploenchan Chetchotsakd, Department of Medicine, Srinagarind Hospital. Tel: 043 363664 or Mobile phone: 01 5925907
2. Dr Piroon Mootsikapun, Department of Medicine, Srinagarind Hospital. Tel: 043 363664 or Mobile phone: 01 8721858
3. Dr Siriluck Anunnatsiri, Department of Medicine, Srinagarind Hospital. Tel: 043 363664 or Mobile phone: 01 0511995

Sappasithiprasong Hospital

1. Dr Direk Limmathurotsakul: Melioidosis Laboratory, Sappasithiprasong Hospital. Tel: 045 246112, Mobile phone: 09 7543784
2. Dr Atchriya Hemachandra: Melioidosis Laboratory, Sappasithiprasong Hospital. Tel: 045 246112, Mobile phone: 06 5887665
3. Prof Wipada Chaowagul: Medical Department, Sappasithiprasong Hospital. Tel: 045 246112, Mobile phone: 01 8765372
4. Dr Wirongrong Chierakul: Melioidosis Laboratory, Sappasithiprasong Hospital. Tel: 045 246112 or Faculty of Tropical Medicine, Mahidol University: 02 3541395 or Mobile phone: 09 1058571

Khon Kaen Hospital

1. Dr Wattanachai Susaengrat, Medical Department, Khon Kaen Hospital. Tel: 043 236789 Ext 1174 or Mobile phone: 01 5444860
2. Dr Sakesan Chaisuksan, Medical Department, Khon Kaen Hospital. Tel: 043 236789 Ext 1174 or Mobile phone: 01 5440233

Udon Thani Hospital

1. Dr.Sunee Chueasuwanchai, Wellcome Laboratory, Udon Thani General Hospital. Tel: 042 347733 or Mobile phone: 01 8622202
2. Dr.Kriangsak Pimda, Medical Department, Udon Thani General Hospital. Tel: 042 244272 (Ext. 7162) or Mobile phone: 01 8717573

3. Dr Sunchai Piphitaporn, Medical Department, Udon Thani General Hospital. Tel: 042 244272 (Ext. 7162) or Mobile phone: 01 3205186

4. Dr Wirongrong Chierakul: Leptospirosis Laboratory, Udon Thani General Hospital. Tel: 042 347733 or Faculty of Tropical Medicine, Mahidol University: 02 3541395 or Mobile phone: 09 1058571

Mahasarakam Hospital

1. Dr Chalongchai Toondee, Mahasarakam Hospital Tel: 043 740937-9 or Mobile phone: 09 4229030

2. Dr Jiraporn Pilaikul, Mahasarakam Hospital Tel: 043 740937-9 or Mobile phone: 01 6614353

I have read the document and hereby agree to participate in this research study.

.....Patient

.....Researcher

.....Witness

.....Witness

I cannot read, but the researcher has read the document for me. I have listened and clearly understand. I hereby agree to participate in this research study.

.....Patient

.....Researcher

.....Witness

.....Witness

In case of age under 18, the guidance or parents must be consented and sign.

.....Guidance/Parents
